# Identification of Claudin 1 Transcript Variants in Human Invasive Breast Cancer

**DOI:** 10.1371/journal.pone.0163387

**Published:** 2016-09-20

**Authors:** Anne A. Blanchard, Teresa Zelinski, Jiuyong Xie, Steven Cooper, Carla Penner, Etienne Leygue, Yvonne Myal

**Affiliations:** 1 Department of Pathology, University of Manitoba, Winnipeg, Manitoba, Canada; 2 Department of Physiology and Pathophysiology, University of Manitoba, Winnipeg, Manitoba, Canada; 3 Department of Pediatrics and Child Health, University of Manitoba, Winnipeg, Manitoba, Canada; 4 Department of Biochemistry and Medical Genetics, University of Manitoba, Winnipeg, Manitoba, Canada; 5 Manitoba Institute of Cell Biology, Winnipeg, Manitoba, Canada; Ohio State University Wexner Medical Center, UNITED STATES

## Abstract

**Background:**

The claudin 1 tight junction protein, solely responsible for the barrier function of epithelial cells, is frequently down regulated in invasive human breast cancer. The underlying mechanism is largely unknown, and no obvious mutations in the claudin 1 gene (*CLDN1*) have been identified to date in breast cancer. Since many genes have been shown to undergo deregulation through splicing and mis-splicing events in cancer, the current study was undertaken to investigate the occurrence of transcript variants for *CLDN1* in human invasive breast cancer.

**Methods:**

RT-PCR analysis of *CLDN1* transcripts was conducted on RNA isolated from 12 human invasive breast tumors. The PCR products from each tumor were resolved by agarose gel electrophoresis, cloned and sequenced. Genomic DNA was also isolated from each of the 12 tumors and amplified using PCR *CLDN1* specific primers. Sanger sequencing and single nucleotide polymorphism (SNP) analyses were conducted.

**Results:**

A number of *CLDN1* transcript variants were identified in these breast tumors. All variants were shorter than the classical *CLDN1* transcript. Sequence analysis of the PCR products revealed several splice variants, primarily in exon 1 of *CLDN1*; resulting in truncated proteins. One variant, V1, resulted in a premature stop codon and thus likely led to nonsense mediated decay. Interestingly, another transcript variant, V2, was not detected in normal breast tissue samples. Further, sequence analysis of the tumor genomic DNA revealed SNPs in 3 of the 4 coding exons, including a rare missense SNP (rs140846629) in exon 2 which represents an Ala124Thr substitution. To our knowledge this is the first report of *CLDN1* transcript variants in human invasive breast cancer. These studies suggest that alternate splicing may also be a mechanism by which claudin 1 is down regulated at both the mRNA and protein levels in invasive breast cancer and may provide novel insights into how *CLDN1* is reduced or silenced in human breast cancer.

## Introduction

Tight junctions (TJs) serve a crucial role in maintaining homeostasis and the cell-cell interactions between epithelial cells. These junctional stuctures block the free diffusion of lipids and proteins by regulating the flow of small ions between cells [1–3]. TJs also serve an important role in maintaining epithelial cell architecture and retaining apical-basolateral polarity [4].

Claudin 1 forms the backbone of TJs, and has been shown to be solely responsible for the epithelial cell barrier function, i.e. separating the internal milieu from the external environment [5]. In cancer, the role of claudin 1 appears to be dichotomous; upregulated in some cancers [6–12] and downregulated in others [13–16]. Moreover, claudin 1 has recently been shown to promote cancer progression and metastasis in several cancers including colon, liver and squamous cell carcinomas [7,10,17]. In breast cancer, its role remains a bit of an enigma. Suggested to be a tumor suppressor, down regulation of the claudin 1 gene (*CLDN1)* has been associated with estrogen receptor (ER) positivity [18] and poor prognosis in invasive breast cancer [15]. However, high levels of claudin 1 have recently been demonstrated in a small but significant group of breast cancers, including the largely aggressive basal-like subtype [18].

The mechanisms by which *CLDN1* is regulated in breast cancer are largely unknown. To date, no *CLDN1* mutations have been identified either through promoter sequencing or exome sequencing analyses. However, a search of The Cancer Genome Atlas (TCGA) database (a large human invasive breast cancer cohort), revealed *CLDN1* was one of a group of highly methylated genes [19], suggesting that epigenetic factors may also play a significant role in the regulation of this gene in some breast cancer subtypes.

Recently, genome-wide analyses have revealed that as many as 95% of human genes undergo alternative splicing [20] and that mis-splicing events (mis-regulation) are often related to cancer and other human diseases. Alternative splicing is known to give rise to transcript variants and can result in the encoding of different protein isoforms. *CLDN1* is located at 3q28-q29 and is comprised of four exons. It has one classical transcript (3.4kb), which codes for a functional 21kD protein [21]. A second non-coding transcript has also been identified, and does not give rise to a translated protein product [21]. In the present study, we investigated whether *CLDN1* transcript variants occur in human invasive breast cancers. We have identified a number of *CLDN1* transcript variants, and propose that other epigenetic factors such as transcript variant utilization may be another mechanism by which *CLDN1* is regulated in breast cancer.

## Materials and Methods

### Human breast tissue

Twelve human invasive breast cancers used in the current study were obtained from the Manitoba Breast Tumour Bank (MBTB, Department of Pathology, University of Manitoba), which operates with the approval from the Faculty of Medicine, University of Manitoba, Research Ethics Board. Studies were carried out with the approval of the Bannatyne Campus, University of Manitoba, Research Ethics Board. Written informed consent was obtained from all participants. Collection, handling and histo-pathological assessment of tumor tissues have been previously described [22,23]. Six normal breast tissue samples, derived from reduction mammoplasty procedures on healthy women, were also obtained from the MBTB.

### RNA extraction and sequencing of transcripts

Total RNA was extracted from 12 invasive human breast tumors and subjected to RT-PCR analysis using primers flanking the coding region of *CLDN1* (Table 1). Total RNA was extracted from frozen tumor tissue samples using the RNAeasy kit (Qiagen Corporation, Mississauga, Canada) and quantitated spectrophometrically using a NanoDrop 2000 (ThermoFisher Scientific, Waltham, MA, USA). RNA was reverse transcribed using the iScript cDNA synthesis kit (Bio-Rad Laboratories Inc., Mississauga, Canada) according to the manufacturer’s instructions. PCR amplification reaction mixtures contained 50ng of reverse transcribed total RNA, 20mM Tris-HCl, pH 8.4, 50mM KCl, 2.5mM MgCl_2_, 0.2uM of forward and reverse primers (Table 1A), 0.2mM each dNTP, and 1U of Platinum Taq DNA polymerase (Life Technologies, Burlington, Canada). The cycle profile consisted of denaturation at 94°C for 4 min., followed by 50 cycles of 94°C for 30s, 55°C for 30s, and 72°C for 45s and a final extension step of 72°C for 5 min. The PCR products were analyzed by 1% agarose gel by electrophoresis and stained with RedSafe nucleic acid staining solution (iNtRON Biotechnology, FroggaBio, Toronto, Canada). Bands selected for sequencing were excised from the gel and purified using the QIAquick Gel Extraction kit (Qiagen Corporation) and subcloned using the TOPO TA cloning kit (Life Technologies). Sequencing of the PCR product positive clones was conducted at The Centre for Applied Genomics (TCAG, Toronto, Canada).

**Table 1:** Primer sequences.

A. Primers used to amplify the full length coding regions of claudins 1, 3 and 4.			
	Forward (5’-3’)	Reverse (5’-3’)	PCR product size (bp)
*CLDN1*	GAGCGAGTCATGGCCAAC	TCTCAATGTCCATTTTCGGTTT	700
*CLDN3*	CGCGGCAGCCATGTCCAT	GGTGGTGGTGGTGGGGTCTCC	706
*CLDN4*	CGTGGACGCTGAACAATG	TCAGTCCAGGGAAGAACAAAG	695
B. Primers designed to amplify the exon coding regions of the *CLDN1* gene^1^			
Exon1	GCTCCCCGCCTTAACTTC	agagcacatgatcagaagacttg	478
Exon2	tggggtctctagattcctagtcc	caggtctatgtttgcagtttgc	414
Exon3	tctggacttctaatctccctaatacc	tcagagatcatttaaattgtttgtagg	381
Exon4	ttcccaagggatattcaggg	TTAAGCCATGTTTAGCACTGAG	471
C. Primers designed to amplify the transcript variants (V)			
V1	GCCCAGGCCATGCACATT	TCTCAATGTCCATTTTCGGTTT	479
V2	TACTCCTATGCCGGCGATATTT	TCTCAATGTCCATTTTCGGTTT	365
V3	GAGCGAGTCATGGCCAAC	GGACAGGAACAGCAAAGAGTCA	231

^1^Note: capital letters indicate that the primer is in the exon sequence, small letters indicate that the primer is in the intron sequence.

### Genomic DNA extraction and sequencing of exon coding regions

Genomic DNA was extracted from frozen tissue by overnight digestion at 55°C with Proteinase K (Roche Diagnostics, Indianapolis, IN, U.S.A.) followed by phenol/chloroform extraction. The DNA concentration was measured spectrophometrically using a NanoDrop 2000 (ThermoFisher Scientific). PCR amplification of the exon coding regions was conducted using 150ng of genomic DNA, using 1U of Platinum Taq DNA polymerase and 0.2uM of forward and reverse primers (Table 1B). The cycle profile consisted of denaturation at 94°C for 5 min., followed by 45 cycles of 94°C for 45s, 58°C for 30s, and 72°C for 1 min. and a final extension step of 72°C for 5 min. The PCR products were analyzed by 1% agarose gel electrophoresis and bands were excised and purified from the gel using the QIAquick Gel Extraction kit (Qiagen Corporation). Sequencing of the purified products was conducted at The Centre for Applied Genomics (TCAG, Toronto, Ontario).

### Identification of transcript variants and SNPs

Clustal Omega [24] was used to align the sequences of the transcript variants with the human *CLDN1* mRNA sequence from GenBank (NM_021101.4), and the sequences from the exon coding regions with the *CLDN1* gene sequence from Ensembl (ENSG00000163347, [21]). Sequence variants were matched with their dbSNP reference SNP identifier (http://www.ncbi.nlm.nih.gov/SNP).

### Immunohistochemistry (IHC)

Tumor sections (5 μm) were cut from paraffin-embedded tissue, mounted on Fisherbrand Superfrost/plus slides (ThermoFisher Scientific) and stained with the rabbit polyclonal claudin 1 antibody (Life Technologies) at a dilution of 1:150. This antibody has been previously validated for specificity and sensitivity in our laboratory [18]. IHC was performed as recently reported [25] using an automated tissue immunostainer (Discovery Staining Module, Ventana Medical Systems, AZ, USA). Briefly, sections were dewaxed in two xylene baths, taken through a series of alcohols, rehydrated and then submitted to heat-induced antigen retrieval for 8 min in the presence of a citrate buffer (CC1, Ventana Medical Systems) using the automated tissue immunostainer (Discovery Staining Module, Ventana Medical Systems). Primary antibodies were applied for 60 min and secondary antibodies for 32 min.

Positive staining for claudin 1 protein was assessed using semi-quantitative scoring (H-scores). H-scores were derived from both staining intensity (scale 0–3) and percentage of positive cells (0–100%), which when multiplied, generated a score ranging from 0–300. Tissue microarray (TMA) staining was evaluated independently by two investigators (AB, CP) and where discordant, cases were re-evaluated jointly.

### Statistical Analysis

Frequency of SNPs in human breast tumors versus the normal population was analysed using the Fisher’s Exact Test (GraphPad Prism version 6.07, GraphPad Software, La Jolla California USA).

## Results

### Identification of *CLDN1* transcript variants in human invasive breast cancer

**Fig 1:**
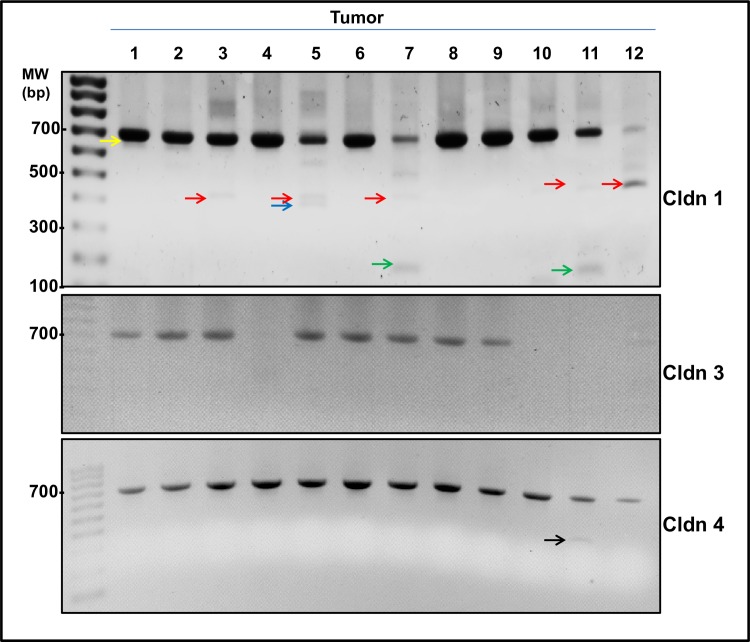
Identification of *CLDN1* transcript variants in invasive human breast cancer. PCR analysis was carried out on reverse transcribed RNA from 12 breast tumors using primers (Table 1) flanking the coding regions of *CLDN1*, *3*, and *4*. The expected full length cDNA products (representing the classical transcripts) for *CLDN1* and *CLDN4* (700 bases and 706 bases respectively) was evident in all tumors. However, no *CLDN3* transcript was detected in some of the breast tumors (middle panel; lanes 4, 10, 11). Colored arrows indicate *CLDN1* PCR products which were verified by Sanger sequencing: yellow arrow, transcript variant 1 (V1) is 615 bp; red arrow, transcript variant 2 (V2) is 440bp; blue arrow, transcript variant 3 (V3) is 362 bp; and green arrow, transcript variant 4 (V4) is 217 bp. In the lower panel, the product indicated by the black arrow, was a non-specific band and had no sequence homology to *CLDN4*.

**Fig 2:**
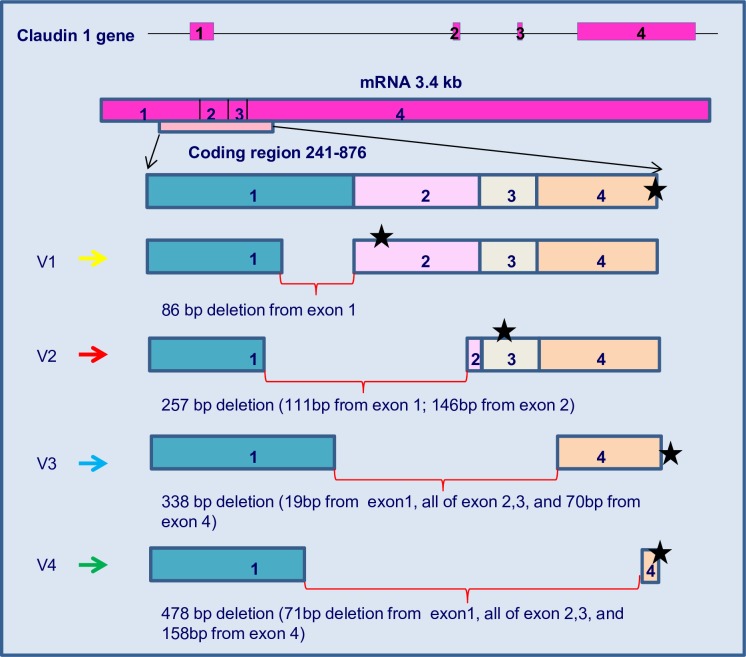
*CLDN1* transcript variants reveal alternate splicing within exons. The shorter *CLDN1* transcript variants were aligned with the full length coding region (636 bp). Translation of the putative protein of each variant resulted in alignment only in the N-terminal of claudin 1 (exon 1). Remaining sequence was out of frame and translated into a non-functional claudin 1 protein. * = location of stop codon.

To investigate *CLDN1* transcript variants in human invasive breast cancer, RT-PCR analysis was performed on breast tumor samples as described in the methods section. In addition to the full length transcript, a number of smaller PCR products were observed in several tumor samples. A representative result is depicted in Fig 1. In multi-template PCRs, rare templates can often be diluted out, or template concentration can be biased by differences in amplification efficiency [26]. Thus, it was not possible to gel purify sufficient quantities of DNA in order to clone and sequence all products. However, as proof of principle, sequencing of some PCR products revealed several shorter cDNA products (Fig 2). Four were selected for further investigation. These were verified as *CLDN1* transcript variants. The largest PCR product, V1 (representing the largest *CLDN1* transcript variant), was the result of an alternate splice site at the 3’ end of exon 1 but which exhibited normal splicing events at exon 2 (Fig 2). Another, designated *CLDN1* transcript variant, V2, displayed alternate splice sites in exon 1 and 2, while alternate splice sites in exons 1 and exon 4 were identified in the two other transcript variants, V3 and V4 (Fig 2). Interestingly, the V1 results in premature stop codons and is expected to be degraded through nonsense-mediated decay. Thus, this perhaps contributed to the relatively lower level of the claudin 1 full length transcript in tumors #5, 7, 11 and 12 (Fig 1).

To determine whether transcript variants were a common occurrence among members of the claudin protein family, particularly among those members which have been shown to be up regulated in breast cancer, claudin 3 and claudin 4 [27,28], RT-PCR analysis was carried out. Only the expected, full length PCR products for both of *CLDN3* and *CLDN4* (706 bp for *CLDN3*, and 695 bp for *CLDN4*) were identified, with the exception of a non-specific product for *CLDN4* which, when sequenced, lacked any sequence homology to *CLDN4* (Fig 1).

### Expression of transcript variants in normal human breast tissue and breast tumors

**Fig 3:**
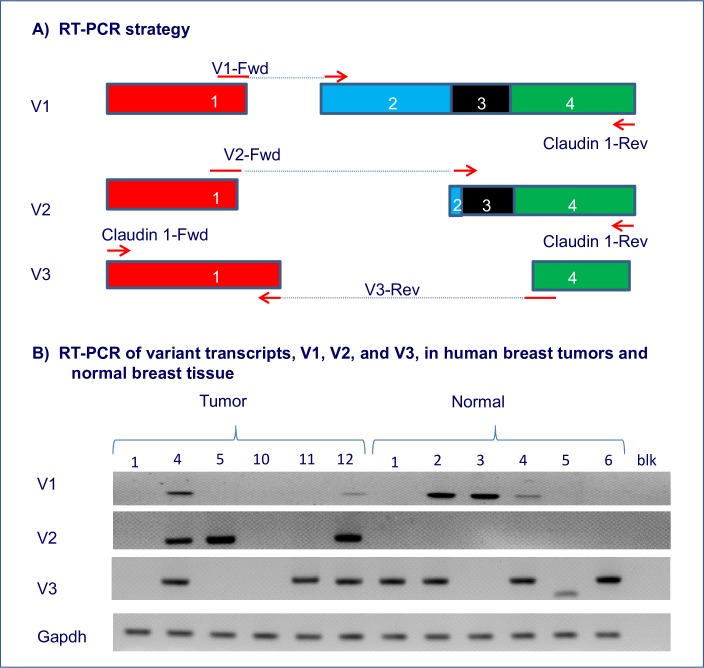
*CLDN1* transcript variants in human breast tumors versus normal breast. **A. Primer Design.** Forward primers were designed to specifically amplify V1 and V2 *CLDN1* transcripts. The reverse primer flanked the coding region of *CLDN1* mRNA and was identical to the one used for the PCR amplification shown in Fig 1. A reverse primer was specifically designed to amplify the V3 transcript variant and used in conjunction with the *CLDN1* forward primer (Table 1). **B. Differential expression of transcript variants.** V1 was amplified in both breast cancer (2/6 tumors) and normal tissue samples (3/6). However, V2 was only evident in the breast tumors (3/6). V3 was detected in both tumors (3/6 tumors) and normal tissue (4/6). Gapdh was used as a control for the RT-PCR reaction. (blk = no template).

To determine whether transcript variants were expressed in normal breast tissue samples, primers were designed to amplify three *CLDN1* transcript variants, V1, V2 and V3. PCR analysis was conducted on six normal breast tissue samples and six randomly selected breast tumor samples (Fig 3). *CLDN1* transcript variant, V1, was amplified in breast cancer tissue (2/6 tumors) as well as normal human breast tissue samples (3/6). Transcript variant, V3, was detected in both breast cancer tissue (3/6 tumors) and in normal human breast tissue samples (4/6). However, V2 was present only in breast tumor samples (3/6). This transcript variant was absent in the normal breast tissue samples.

PCR analysis was performed on the six remaining tumors, however only four were evaluated as the material for two were exhausted. V1 was amplified in a total of 4/10 tumors, V2 in 5/10 tumors, while V3 was amplified in 6/10 tumors (Table 2).

**Table 2:** Clinico-pathological characteristics and claudin 1 protein levels in human breast tumors. ER α and PR status, determined by the ligand binding assay (ER+, >3fmol/mg protein; PR+, >10fmol/mg protein), tumor size, grade and nodal status were obtained from the Manitoba Breast Tumor Bank. Claudin 1 protein levels were evaluated by immunohistochemistry. H scores: % claudin 1 positive tumor cells multiplied by the intensity of staining (scale 1–3). H scores were further categorized as 0–15 = —(neglible); 20–75 = + (low); 80–150 = ++ (moderate); 155–300 = +++ (high). There was a trend towards high claudin 1 levels (3/4) being associated with ER- tumors, as previously described [18], but no direct correlation with transcript variant and ER status was evident.

Tumor #	Estrogen Receptor (fmol/mg protein)	Estrogen Receptor Status	Progesterone Receptor (fmol/mg protein)	Progesterone Receptor Status	Tumor Size (cm)	Nodal status	Grade	SNP	Transcript Variant expression			Claudin 1 IHC
									V1	V2	V3	
1	92	+	105	+	3.0	+	7	N.D.	-	-	-	-
2	140	+	52.0	+	5.0	+	5	rs3172404	+	+	+	+
3	13.4	+	15.7	+	5.0	+	7	N.D.	-	-	+	-
4	180	+	14.6	+	5.0	-	6	rs140846629	+	+	+	+++
5	12.1	+	12.0	+	6.0	N/A	6	rs72466472	-	+	-	-
6	13.6	+	5.6	-	5.8	-	7	rs9869263	N/A	N/A	N/A	++
7	1	-	4.6	-	7.0	+	6	N.D.	N/A	N/A	N/A	-
8	1.3	-	9.6	-	N/A	+	7	N.D.	-	-	-	+++
9	1	-	2.5	-	5.0	+	8	rs3172404	+	+	+	+
10	12.5	+	89.0	+	4.2	-	5	N.D.	-	-	-	-
11	0	-	20.0	+	8.0	-	7	rs72466472	-	-	+	+++
12	0	-	10.8	+	5.1	+	5	rs3172404; rs17429833	+	+	+	+++

N.D = not detected; N/A = not analyzed.

### Identification of SNPs in *CLDN1*

**Fig 4:**
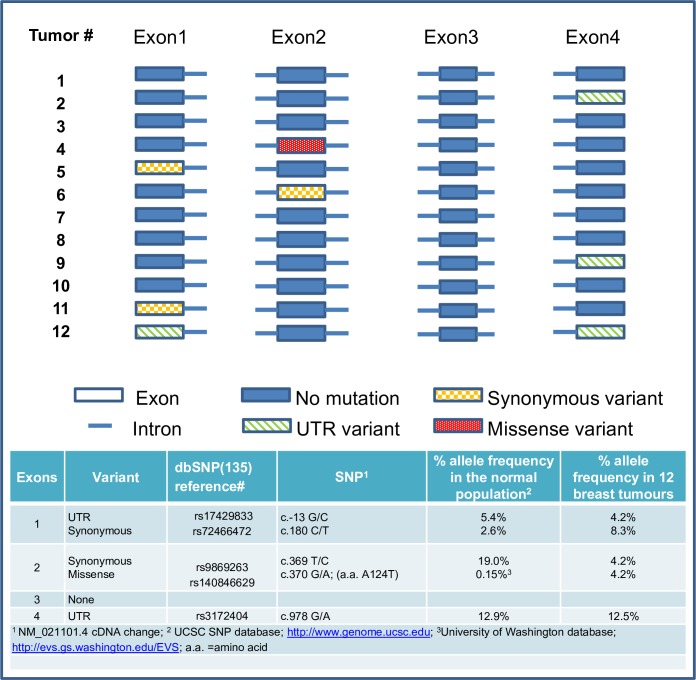
Identification of SNPs in human breast cancers within *CLDN1* exons. Several SNPs were identified by sequencing of the PCR products (see Table 1). The cDNA position of the SNPs are shown and designated by their corresponding Reference SNP (rs) numbers. The frequency of the missense SNP detected in exon 2, was significantly higher in the breast tumors than predicted for the normal population (p< 0.5; two-tailed Fisher’s Exact Test).

To determine whether the transcript variants were a consequence of genomic DNA deletions, PCR analyses were conducted on each of the four *CLDN1* exons using genomic DNA from each of the 12 tumors as templates. Sequence analysis of the PCR products failed to identify any deletions (not shown). However, previously identified heterozygous SNPs were identified (Table 2) in some cases. The SNP (rs17429833), located in the 5’ untranslated region, and identified in only one of the 12 breast cancer specimens (tumor #12, Fig 4) was found to be present in 5.4% of the normal population (University of California Santa Cruz, UCSC, database). A second SNP (rs3172404) was identified in the 3’ untranslated region of the same tumor as well as in two additional tumors (tumors #2, #9; Fig 4). The frequency of occurrence of this SNP in the tumors was calculated to be equivalent to that for the normal population. However, a synonymous SNP (rs72466472) detected in exon 1 of two tumors (tumor #5, #11) exceeded the normal frequency of occurrence (8.3% vs 2.6%; UCSC database) found in the normal population (Fig 4). Conversely, a second synonymous SNP (rs9869263) was identified in exon 2, at a frequency very much below (4.2%) that of the normal population (19%). Of notable interest was the identification of a rare missense variant (rs140846629) in exon 2 of tumor #4. The frequency of this allele (4.2%) was significantly higher (p<0.05) than that calculated for the normal population (0.15%, UCSC SNP database).

### Immunohistochemical analysis of claudin 1 protein in breast tumors

**Fig 5:**
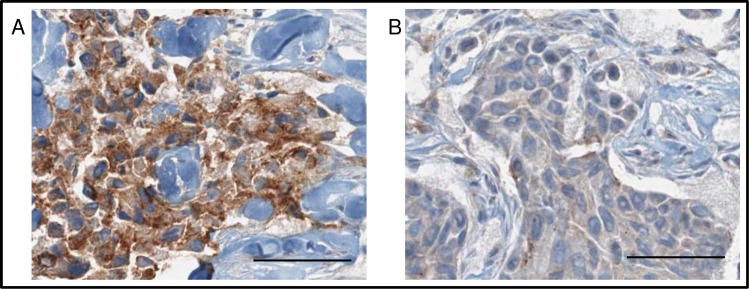
Representative immunostaining of breast tumors with claudin 1 antibody. A human breast tumor exhibiting high (A; tumor #11) and one exhibiting low (B; Tumor #3) levels of the claudin 1 protein. Scale bars = 60 μm.

Next we wanted to examine whether there was a relationship between claudin 1 protein level in the breast cancer specimens and the presence of transcript variants or SNPs. To determine whether claudin 1 protein levels were associated with the presence (or absence) of *CLDN1* transcript variants, immunohistochemical (IHC) analysis of breast tumors was determined using a c-terminal claudin 1 polyclonal antibody. Positive staining was reported by an H score, which incorporated the signal intensity and the number of claudin 1 positive cells [intensity (0–3) multiplied by the % of positive tumor cells] (Table 2 & Fig 5). Results from this analysis revealed that claudin 1 protein levels in the 12 breast tumors ranged from not detectable or low (5/12 tumors) to very high (4/12 tumors). However, though no relationship between the claudin 1 protein levels and the presence of transcript variants or SNPs was overtly evident, tumors #5 and 7, which also expressed low levels of mRNA (Fig 1), showed negligible staining of the claudin 1 protein by IHC.

## Discussion

The deregulation of *CLDN1* in different cancers is not well understood. Claudin 1 has been clearly demonstrated to directly promote carcinogenesis in some cancers [10,29–32]. In others, such as esophageal squamous cell carcinoma [33], lung [34], prostate [35], melanocytic neoplasia [36] and breast cancers [28,37], claudin 1 appears to demonstrate tumor suppressor activities. Further, in breast cancer, a down regulation of *CLDN1* has more frequently been associated with invasiveness and poor patient prognosis [15]. However, an up regulation of *CLDN1* in specific breast cancer subtypes, including the highly aggressive basal-like breast cancer, has now been demonstrated by us and others [18,25,38,39]. The mechanism by which *CLDN1* is regulated in breast cancers is largely unknown. Yet, to date, no mutations have been identified that can be attributed to the silencing of this gene during breast cancer progression.

It is now well recognized that the effect of the genome and epigenome can strongly influence each other in cancer, and that SNPs, transcript variants and methylation are important factors in the regulation of several cancers [40]. It is also recognized that multiple modes of alternative splicing exist, such as alternative 5′ or 3′splice-site usage, as well as differential inclusion or skipping of particular exons, which result in transcript variants. It has been shown that >20,000 single nucleotide variants can disrupt splicing, and that nonsense, missense and synonymous exonic variants can also affect alternative splicing, some as far as 300 base pairs from the splice sites [41]. Furthermore, epigenetic factors play an important role in therapeutic responses as demonstrated by the altered responses to estrogen therapy attributed in part to transcript variants and SNPs [42,43] in some breast cancer patients.

The 17 kb *CLDN1* gene codes for the classical 3.4kb transcript which translates into the intact functional protein. It also gives rise to a non-coding transcript with intron retention [21]. Several (over 80) spliced expressed sequence tags (ESTs) have also been identified (University of California, Santa Cruz (UCSC) Genome browser [44]). In this study, we report for the first time, numerous *CLDN1* transcript variants in breast cancer. Each *CLDN1* transcript variant identified was shorter in length than the expected cDNA product (700bp). Comparison analysis with other members of the claudin family, *CLDN3* and *CLDN4*, whose genes are overexpressed in breast cancer [27,28], revealed that the transcript variants were unique to *CLDN1*. We selected four of the transcript variants (V1, V2, V3, and V4) for further analysis. The splicing sites and patterns identified in these variants were novel and were not, to our knowledge, found to be previously reported in any existing available database. Moreover, two of the variants were identified in some normal tissue as well, suggesting the possibility that the presence of these variants may predispose a person to disease. However, future studies are necessary to show such a causal link. Intriguingly, we observed that one of the transcript variants, V2, was only detected in tumor samples and not in any of the normal breast tissue. As well, a follow-up of the potential significance and consequences of the presence of this transcript variant in breast cancer is warranted. Unfortunately, at this time verification of any putative proteins generated from these transcripts has not been possible due to the lack of commercial antibodies to the N-terminus region of claudin 1. In addition, any proteins resulting from the translation of transcript variants due to deletions could be missed due to mis-match with the c-terminal classical claudin 1 antibody.

A number of *CLDN1* SNPs were also identified in the breast tumor specimens. The synonymous SNP, rs9869263, that we identified in exon 2 in the present study, was reported to occur at a frequency of 2.6% of alleles in the normal population [44]. In the 12 breasts cancers analyzed, 2/12 or 17% of tumors (8.3% of alleles) were positive for this SNP. This is not the first time this SNP has been associated with cancer. The rs9869263 genotype (c.369C>T) was recently shown to be related to differentiation and tumor stage and increased risk of colon cancer [45]. Another SNP, the rs17501976 polymorphism in *CLDN1* was also shown to impact colon cancer risk. However, in the latter case it was shown to be significantly associated with a decreased susceptibility to colorectal cancer in a Chinese population [46].

A search of the Ensembl database revealed nearly 3000 *CLDN1* genetic variants, with over 900 within the gene sequence [21], and some of these SNPs associated with disease. For example, one such SNP, rs9290927, was recently shown to enhance the development of a blood brain barrier disorder [47]. Others, found in the *CLDN1* promoter region, have been shown to confer susceptibility to hepatitis C virus infection [48]. Interestingly, with regard to *CLDN1* SNPs in breast cancer, to date, only one mutation, p.V85G, has been identified in one of 993 patient samples analyzed in the Cancer Genome Atlas project [49].

A c.370G>A mutation, which resulted in the p.A124T substitution, and which was identified in only one out of the twelve breast cancer tumor specimens analyzed, was viewed as a rare polymorphism (rs140846629; frequency of 0.0015), as calculated in both the Ensembl and the Washington database [21,50]. Notably, this amino acid change has been predicted to be a possibly damaging substitution (based on the assigned PolyPhen 2 score of 0.542 [51]), not specifically only in breast cancer but in other cancers as well. Even more intriguing was the observation that the A124T substitution was the only mutation detected in the *CLDN1* gene in 1 of 169 brain lower grade glioma, and in 1 of 132 stomach adenocarcinoma cases, but not in over 900 breast cancers (The Cancer Genome Atlas datasets, [49]). Since we identified this A124T mutation in one in 12 breast tumor samples, it is possible that this variant may occur more frequently in our patient cohort and is important to disease development within this population. As well, our analyses suggest that there is a relationship between the SNP, rs3172404, and the presence of the transcript variants, V1, V2, and V3 in tumors (Table 2, tumors 2, 9, 12). However, whether this SNP is related to the generation of these transcript variants will need to be followed up.

We further investigated whether there was an association between ER positivity and transcript variant expression within this cohort, since our previous work showed a relationship between ER status and claudin 1 levels [18]. Our analysis did not reveal any significant correlation between the presence of the claudin 1 transcript variants and ER status. However, this lack of correlation could merely be attributed to the small sample size, which limited the ability to conduct rigid statistical analyses.

Recent reports suggest that hypermethylation may be a partial mechanism of *CLDN1* silencing in breast cancer [52]. In particular, this was observed in the luminal-like ER+ breast cancers which exhibits low *CLDN1* expression. We have previously shown *in vitro* that the human breast cancer cell line, MCF7, that displays low endogenous claudin 1 levels was hypermethylated compared to the T47D human breast cancer cell line that expresses high claudin 1 levels [53]. Here, in the present study, we now show that in breast cancer there are numerous *CLDN1* transcript variants suggesting that alternate splicing may be another mechanism of *CLDN1* deregulation. We also observed a trend towards increased transcript variants with the more aggressive ER- tumors, suggesting that variant presence could be related to tumor stage. Altogether, these studies suggest that epigenetic factors may play a role in *CLDN1* regulation in breast cancer. However, these observations warrants a larger follow-up study to determine whether *CLDN1* transcript variants are associated with certain breast cancer subtypes and will allow us to ascertain the potential use of these transcript variants as biomarkers in predicting tumor development and outcome in breast cancer.

## Supporting Information

S1 Fig Sequence alignment of the *CLDN1* transcript variants with the classical *CLDN1* mRNA. The PCR product amplified from the classical *CLDN1* mRNA is shown. Highlighted are the primer sequences used (highlighted text), the starting ATG, and the color coded exon sequences (exon 1, red font; exon 2, blue font; exon 3, black font; and exon 4, green font). The transcript variants, V1, V2, V3 and V4 with the deleted regions indicated, are aligned below the classical transcript.
(TIF)

S2 Fig Sequence chromatogram showing the SNP, rs140846629. The PCR product was amplified using exon 2 specific primers (Table 1) from genomic DNA extracted from tumor #4 and sequenced. The SNP (G/A) is highlighted at position 243.
(TIF)

S3 Fig Sequence chromatogram showing the variant transcript, VT2. The PCR product was amplified using primers to the full length coding region of claudin 1, (Table 1) from tumor #2 mRNA and sequenced. The forward primer sequence is highlighted at position 70. The alternate splicing of exon 1,2 is shown at position 191 (S1 Fig).
(TIF)
