# Supplementary figures and images for: Identification of Claudin 1 Transcript Variants in Human Invasive Breast Cancer
Source: PLoS One. 2016 Sep 20;11(9):e0163387. doi: 10.1371/journal.pone.0163387 (PMC5029943; doi:10.1371/journal.pone.0163387)

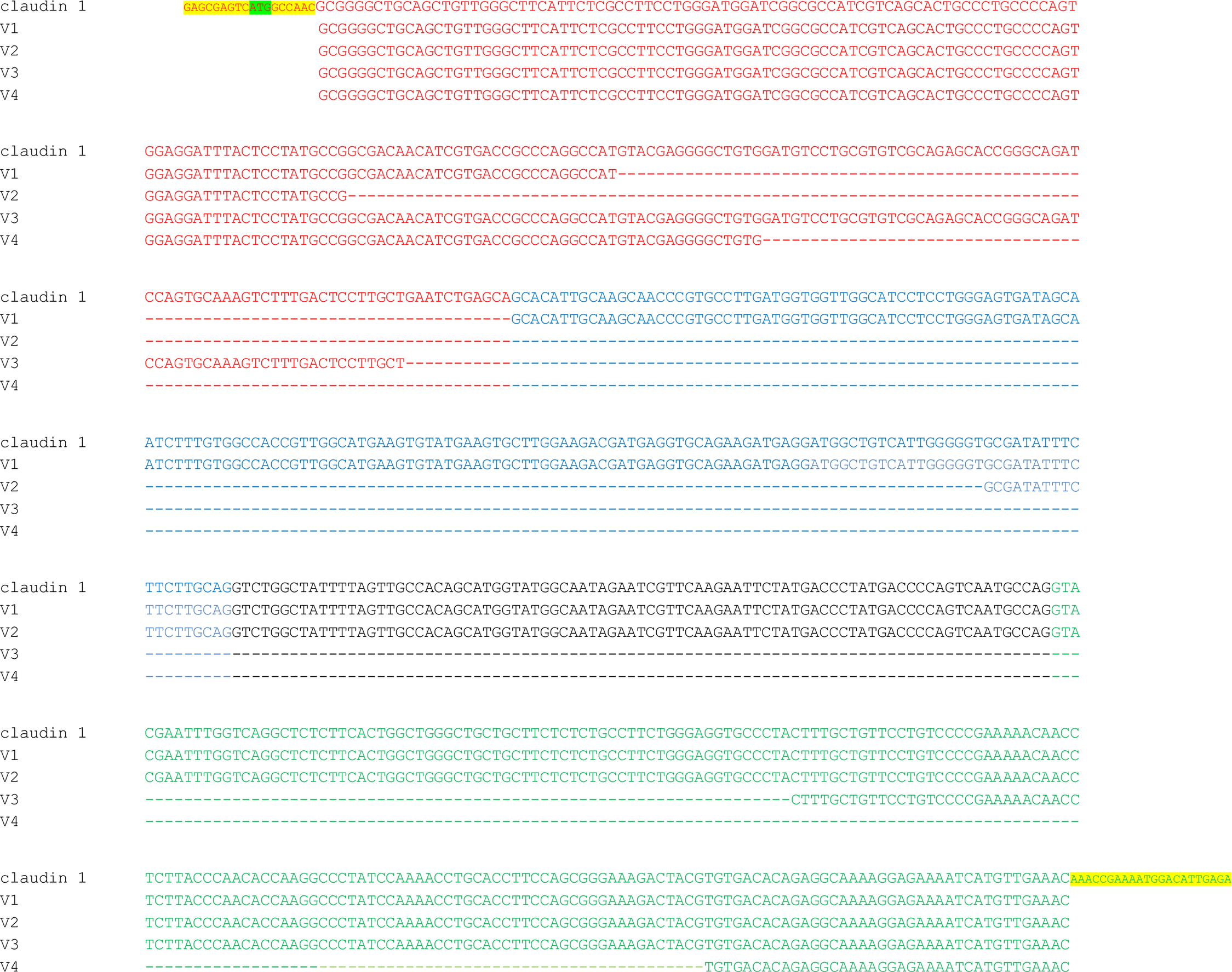

Supplement: S1 Fig — The PCR product amplified from the classical CLDN1 mRNA is shown. Highlighted are the primer sequences used (highlighted text), the starting ATG, and the color coded exon sequences (exon 1, red font; exon 2, blue font; exon 3, black font; and exon 4, green font). The transcript variants, V1, V2, V3 and V4 with the deleted regions indicated, are aligned below the classical transcript. (TIF) [file pone.0163387.s001.tif]

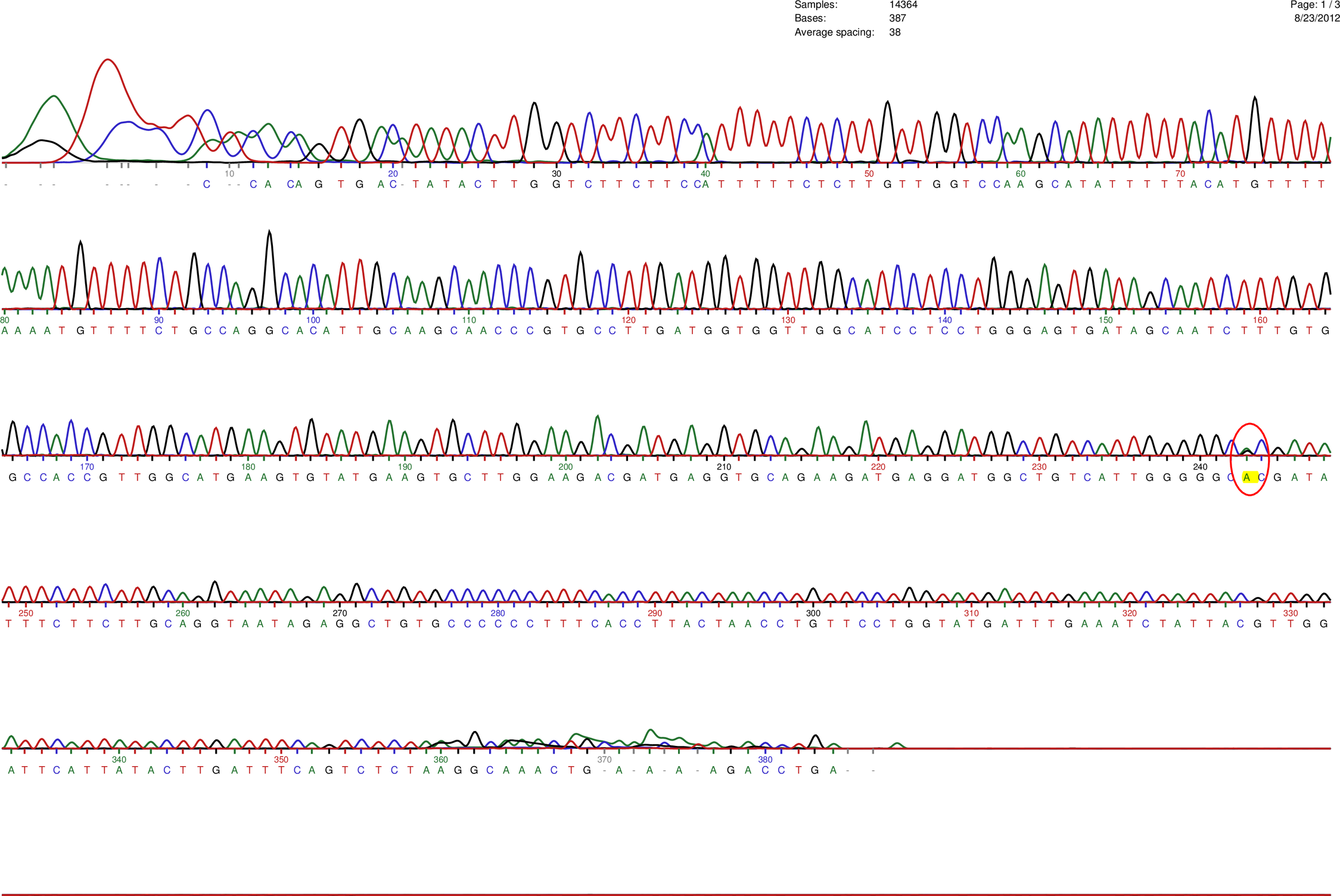

Supplement: S2 Fig — The PCR product was amplified using exon 2 specific primers (Table 1) from genomic DNA extracted from tumor #4 and sequenced. The SNP (G/A) is highlighted at position 243. (TIF) [file pone.0163387.s002.tif]

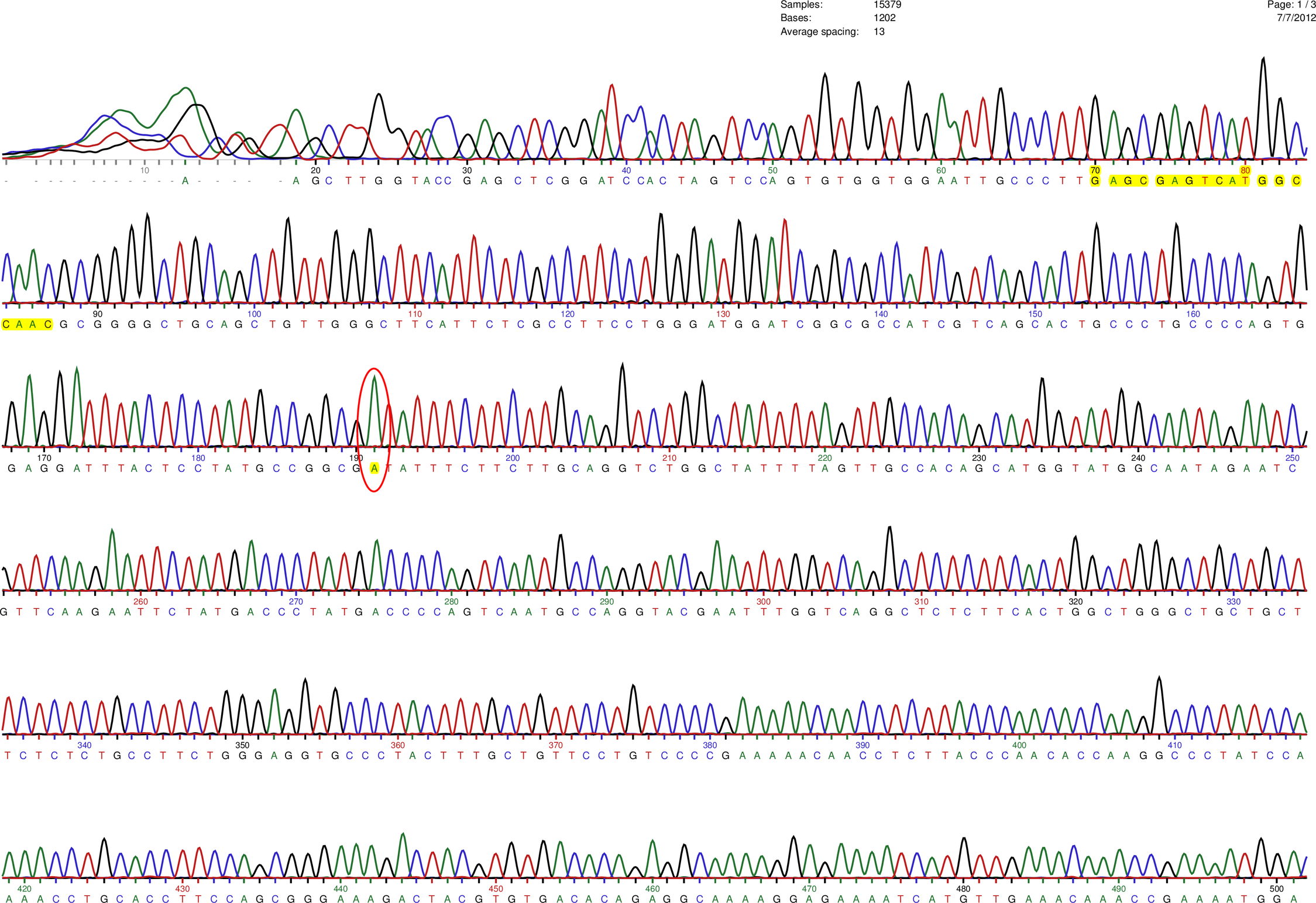

Supplement: S3 Fig — The PCR product was amplified using primers to the full length coding region of claudin 1, (Table 1) from tumor #2 mRNA and sequenced. The forward primer sequence is highlighted at position 70. The alternate splicing of exon 1,2 is shown at position 191 (S1 Fig). (TIF) [file pone.0163387.s003.tif]
